# Oysters as Food

**Published:** 1891-04

**Authors:** 


### OYSTERS AS FOOD.

The *Century Magazine* has this to say regarding the oyster.

Speaking roughly, a quart of oysters contains on the average about the same quantity of active nutritive substance as a quart of milk, or a pound of very lean beef, or a pound and a half of codfish, or two-thirds of a pound of bread. But while the weight of actual nutriment in the different qualities of food named is very nearly the same, the quality is widely different. That of the very lean meat or codfish consists mostly of what are called in chemical language protein compounds or "flesh formers"—the substance which makes blood, muscle, tendon, bone, brain and other nitrogenous tissues. That of the bread contains but little of these, and consists chiefly of starch, with a little fat and other compounds which serve the body as fuel, and supply it with heat and muscular power. The nutritive substance of oysters contains considerable of both the flesh forming and the more especially heat and force giving ingredients. Oysters come nearer to milk than almost any other common food; their values for supplying the body with material to build up its parts, repair its wastes and furnish it with heat and energy would be pretty nearly the same.

### THE FAILURE OF DRUGS.

Unfortunately, the remedies which seem to act beneficially during one epidemic of cholera often prove useless in the next. An eminent Indian medical officer, the late Dr. Norman Chevers, who probably had as great an experience of cholera as any man, stated that the last epidemic of the disease he witnessed was not only different in various characteristics from those he had met with before, but that no treatment satisfactory in other epidemics proved beneficial in the last. To give an account, or even a list, of the various treatments which have been proposed would be a waste of time. From copious bleeding to antimony and purgatives, from calomel to cold water, all have been tried and found wanting. Still, we find medical men extolling some particular line of treatment, or some special drug.

